# *FABP4* Dynamics in Obesity: Discrepancies in Adipose Tissue and Liver Expression Regarding Circulating Plasma Levels

**DOI:** 10.1371/journal.pone.0048605

**Published:** 2012-11-05

**Authors:** María Isabel Queipo-Ortuño, Xavier Escoté, Victoria Ceperuelo-Mallafré, Lourdes Garrido-Sanchez, Merce Miranda, Mercedes Clemente-Postigo, Rafael Pérez-Pérez, Belen Peral, Fernando Cardona, Jose Manuel Fernández-Real, Francisco J. Tinahones, Joan Vendrell

**Affiliations:** 1 Laboratorio de Investigaciones Biomédicas del Hospital Virgen de la Victoria (Fundación IMABIS), Málaga, Spain; 2 CIBER de Fisiopatología de la Obesidad y Nutrición (CIBEROBN), Instituto de Salud Carlos III, Madrid, Spain; 3 Endocrinology and Diabetes Unit.Joan XXIII University Hospital, IISPV, Universitat Rovira i Virgili, Tarragona, Spain; 4 CIBER de Diabetes y Enfermedades Metabólicas Asociadas (CIBERDEM), Instituto de Salud Carlos III, Madrid, Spain; 5 Instituto de Investigaciones Biomédicas, Alberto Sols, Consejo Superior de Investigaciones Científicas (CSIC) & Universidad Autónoma de Madrid (UAM), Madrid, Spain; 6 Diabetes, Endocrinology and Nutrition Service, Institut d’Investigació Biomèdica de Girona (IdIBGi), Girona, Spain; 7 Servicio Endocrinología y Nutrición del Hospital Universitario Virgen de la Victoria, Málaga, Spain; Wageningen University, The Netherlands

## Abstract

**Background:**

FABP4 is predominantly expressed in adipose tissue, and its circulating levels are linked with obesity and a poor atherogenic profile.

**Objective:**

In patients with a wide BMI range, we analyze *FABP4* expression in adipose and hepatic tissues in the settings of obesity and insulin resistance. Associations between *FABP4* expression in adipose tissue and the FABP4 plasma level as well as the main adipogenic and lipolytic genes expressed in adipose tissue were also analyzed.

**Methods:**

The expression of several lipogenic, lipolytic, *PPAR family* and *FABP family* genes was analyzed by real time PCR. FABP4 protein expression in total adipose tissues and its fractions were determined by western blot.

**Results:**

In obesity *FABP4* expression was down-regulated (at both mRNA and protein levels), with its levels mainly predicted by *ATGL* and inversely by the HOMA-IR index. The BMI appeared as the only determinant of the *FABP4* variation in both adipose tissue depots. FABP4 plasma levels showed a significant progressive increase according to BMI but no association was detected between FABP4 circulating levels and SAT or VAT *FABP4* gene expression. The gene expression of FABP1, FABP4 and FABP5 in hepatic tissue was significantly higher in tissue from the obese IR patients compared to the non-IR group.

**Conclusion:**

The inverse pattern in FABP4 expression between adipose and hepatic tissue observed in morbid obese patients, regarding the IR context, suggests that both tissues may act in a balanced manner. These differences may help us to understand the discrepancies between circulating plasma levels and adipose tissue expression in obesity.

## Introduction

Cytoplasmic fatty-acid-binding proteins (FABPs) are proteins with a tissue-specific distribution implicated in cellular uptake and transport of fatty acids as well as coordination of metabolic and inflammatory pathways and modulation of gene expression [1], [2]. FABP4 is highly expressed in adipose tissue and also expressed in macrophages; it is one of the most abundant proteins in mature adipocytes [3] and is also detected at high concentrations in human serum [4], [5]. Although the biological role of FABP4 is not yet well understood, its function has been linked to insulin sensitivity, lipid metabolism and inflammation [6]. Recently, FABP4 has emerged as an important mediator in the crosstalk between adipocytes and macrophages in adipose tissue. Animal studies have shown that FABP4 knock-out mice are protected from the development of obesity-induced IR, impaired glucose tolerance and atherosclerosis, and their adipocytes have reduced lipolysis effectiveness [7], [8]. FABP4 −/− mice in an apo E −/− background show delayed development of arteriosclerotic plaques [9].

Others studies have shown that FABP4 plasma concentrations are increased in patients with obesity, metabolic syndrome (MS), type 2 diabetes (T2D), familial combined hyperlipidemia, lipodystrophy syndromes and cardiovascular disease [10]–[15]. Moreover, there is an increasing evidence based on population studies supporting the predictive role of increased serum FABP4 for MS and cardiometabolic risk. In cross-sectional studies including overweight or moderately obese patients, FABP4 was closely associated with obesity and MS [5], [16]. Furthermore, in others prospective studies FABP4 levels are considered an early marker of metabolic risk for metabolic syndrome development and T2D [4], [17], [18]. These findings suggest that circulating FABP4 could promote inflammation, mediate insulin resistance, type 2 diabetes and atherosclerosis. In addition, FABP4 could be a potential target for treating metabolic diseases [19].

On the other hand, metabolic and functional differences between adipose tissue depots (subcutaneous (SAT) and visceral (VAT)) may be determinant in the development of obesity and related disorders [20], [21]. Some differences in lipogenic, lipolytic and adipokine gene profiles between the two depots have been reported in human studies [22]. Furthermore, several studies have shown a higher gene expression of FABP4 in the SAT than in the VAT of obese subjects [23], [24]. However, other authors failed to find a significant difference between FABP4 gene expression in SAT and VAT [25]. In a recent study that we have carried out, we have found that FABP4 gene expression was significantly lower in the morbidly obese group than in obese and lean subjects in the VAT and in the SAT [26]. However, no data are yet available about FABP4 concerning both adipose tissue depots and the liver. More recently, targeted deletion of certain FABPs in murine models has revealed that FABP4 and FABP5 deficient mice are protected against diet-induced obesity, insulin resistance, type 2 diabetes, and a fatty liver, implying that these proteins may play a role in regulating liver fat [27].

We hypothesized that FABP4 may have an important role in the metabolic balance of the adipose and liver tissue in obesity. The aim of this study was to analyze the *FABP4* expression in paired samples of SAT and VAT and hepatic tissues in the context of obesity and IR in patients with a wide BMI range. We also determined the possible relationships between the SAT and VAT *FABP4* expression and the FABP4 plasma level as well as the main adipogenic and lipolytic genes expressed in adipose tissue.

## Methods and Procedures

### Ethics Statement

#### Human samples

Appropriate Institutional Review Board approval and adequate Biobank written informed consent were obtained from all the participants. The ethics committees of the corresponding hospitals (Joan XXIII University Hospital (Tarragona, Spain) and Virgen de la Victoria Hospital (Malaga, Spain) reviewed and approved the experimental protocols.

#### Animal samples

All animal studies were conducted in accordance with the ethical guidelines for the care and use of laboratory animals of the National Institutes of Health. The protocol was approved by the ethics committee of the Virgen de la Victoria Hospital (Malaga, Spain).

### Processing and Selection of Human Study Samples

Between January 2010 and January 2011, two hundred forty-eight subjects were assessed by the endocrinology and surgery departments at Joan XXIII University Hospital (Tarragona, Spain) and Virgen de la Victoria Hospital (Malaga, Spain). All patients who met the inclusion criteria were recruited to carry out this study. Bio-banking samples included total adipose tissue from subcutaneous and visceral origin, liver tissue, serum and plasma. All the patients had fasted overnight, at least 12 hours before the surgical procedure. Blood samples were collected before the surgical procedure from the antecubital vein, 20 ml of blood with EDTA (1 mg/ml) and 10 ml of blood in silicone tubes. 15 ml of collected blood was used for plasma separation. Plasma and serum samples were stored at −80°C until analytical measurements were performed. EDTA collected blood was used to determine HbA1c. VAT and SAT samples from the same individual were obtained during the surgical procedure. Adipose tissue samples were collected and washed in PBS 1X, immediately frozen in liquid nitrogen and stored at −80°C.

Samples were selected according to stratification by age, gender and BMI (categorized using the World Health Organization criteria) [28]. A first cohort of 62 subjects with a body mass index (BMI) range of 19.82 to 38.54 Kg/m^2^ recruited before cholecystectomy or surgery for abdominal hernia was selected from the Joan XXIII University Hospital biobank. A second cohort of 12 lean subjects (BMI <25) recruited before cholecystectomy and 40 morbidly obese subjects recruited before bariatric surgery were selected for the study from the Virgen de la Victoria Hospital biobank. The morbidly obese subjects were stratified according to their IR index (HOMA-IR) as high-grade IR (MO-IR; HOMA-IR>8,) or non IR (MO non-IR; HOMA-IR<4.7). The cut-off point for the HOMA-IR was taken from previous studies carried out in a healthy population with no carbohydrate metabolism disorders [29].

All the subjects were of Caucasian origin and reported that their body weight had been stable for at least 3 months before the study. They had no systemic disease other than obesity, and all had been free of any infections during the month before the study. Primary cardiovascular disease, arthritis, acute inflammatory disease and renal diseases were specifically excluded by biochemical work-up. Patients on lipid-lowering drugs were excluded from this study. Smoking habits and alcohol consumption were measured using a standardized questionnaire. All the experiments described below were performed blindly.

### Anthropometric Measurements

The height was measured to the nearest 0.5 cm and the body weight to the nearest 0.1 kg. The BMI was calculated as weight (kilograms) divided by height (metres) squared. The waist circumference was measured midway between the lowest rib margin and the iliac crest [30].

### Adipose Tissue Sampling and RNA Extraction

Biopsy samples of VAT (omental) and SAT (anterior abdominal wall) were obtained from all subjects. Samples were obtained during abdominal elective surgical procedures (cholecystectomy, surgery for abdominal hernia or during bariatric surgery). The biopsy samples were washed in physiological saline and immediately frozen in liquid nitrogen. The biopsy samples were maintained at −80°C until analysis. Total RNA isolation from adipose tissues was obtained using RNeasy Lipid Tissue Mini Kit (Qiagen GmbH, Germany) and treated with DNase (RNase-free DNase Set; Qiagen). The RNA concentration was determined by absorbance at 260 nm (A260), and the purity was estimated by determining the A260/A280 ratio with a Nanodrop spectrophotometer (Nanodrop Technologies, Wilmington, DE). The integrity of total purified RNA was checked by denaturing agarose gel electrophoresis and ethidium bromide staining.

### Liver Biopsy and Total RNA Extraction

In the morbidly obese cohort, wedge biopsies (10–400 mg) were taken when necessary during the bariatric procedure for histopathological assessment. Seventeen biopsies were available for the study. The samples were immediately frozen and stored at −80°C. Frozen tissue samples (200–300 mg) were homogenized in 1 ml of Tripure isolation reagent and RNA extraction was done following the manufacturer’s instructions (Roche Applied Science). The RNA concentration and purity were determined in the same way as explained for the adipose tissue above.

### Adipose Tissue Fractionation

Fresh adipose tissue was finely diced into small pieces (10–30 mg), washed in PBS and incubated in Medium 199 (Gibco) plus 4% BSA and 2 mg/mL of collagenase Type I (Sigma-Aldrich) for 1 h in a shaking water bath at 37°C. Mature adipocytes were separated by filtration through a 200µm mesh fabric (Spectrum Laboratories, Rancho Domínguez, CA, USA) and by centrifugation for 5 min at 1500 g. The mature adipocytes were removed from the top layer and the pellet consisted of stromovascular cells. Cells were washed 4 times in PBS. Total RNA from isolated adipocytes and from the stromovascular fraction (SVF) was extracted as in [31].

### Analytical Methods

Serum glucose, cholesterol, HDL cholesterol, triglycerides (Randox Laboratories Ltd., Antrium, UK) and free fatty acids (FFA) (Wako Bioproducts, Richmond, VA, USA) were measured using standard enzymatic methods. Low-density lipoprotein (LDL) cholesterol was calculated using the Friedewald formula. The insulin was analyzed using an immunoradiometric assay (IRMA) (BioSource International, Camarillo, CA, USA), showing a 0.3% cross-reaction with proinsulin. The intra- and inter-assay CV were 1.9% and 6.3%, respectively. The homeostasis model assessment of insulin resistance (HOMA-IR index) was determined as glucose (mmol/L) x insulin (mIU/L)]/22.5 [16]. Plasma FABP4 levels were measured by sandwich enzyme-linked immunosorbent assay (BioVendor Laboratory Medicine, Inc Palackeho, Czech Republic). The sensitivity was 0.2 ng/mL. The intra- and inter-assay coefficients of variation were 5.8% and 14.7%, respectively.

### Gene Expression Relative Quantification in Human Samples

For first-strand cDNA synthesis, a constant amount of 1 µg of total RNA was reverse transcribed using random hexamers as primers and Transcriptor Reverse Transcriptase (Roche, Mannheim, Germany). mRNA levels were assessed by real-time PCR using an Applied Biosystems 7500 Fast Real-Time PCR System (Applied Biosystems, Darmstadt, Germany) with TaqMan technology. The reaction was performed, following the manufacturer’s protocol, in a final volume of 25 µl. The cycle program consisted of an initial denaturing of 10 min at 95°C, then 40 cycles of 15-s denaturing phase at 95°C, and 1 min annealing and extension phase at 60°C. The commercially available and prevalidated TaqMan primer/probe sets used for human samples were as follows: *GAPDH* (4326317E, RefSeq. NM_002046.3) and *β-Actin* (4352935E, RefSeq. NM_001101.2) used as endogenous controls for the target gene in each reaction of samples from liver and adipose tissue, respectively. We analyzed the mRNA expression levels in SAT and VAT of peroxisome proliferator-activated receptors (*PPAR*α, (Hs00947539_m1, RefSeq NM_001001928.2), *PPAR*δ (Hs00234592_m1, RefSeq NM_005037.5) and *PPAR*γ (Hs00234592_m1, RefSeq NM_138712); hormone-sensitive lipase (*HSL)* (Hs00193510_m1, RefSeq NM_005357); adipose triglyceride lipase (*ATGL*) (Hs00386101_m1, RefSeq NM_020376); acyl-CoA synthetase short-chain family member 2 *(ACSS2*) (Hs01120921_g1 RefSeq NM_001242393.1); CD36 molecule *(CD36) (*Hs01567185_m1, RefSeq NM_000072.3)*;* Adiponectin *(APM)* (Hs00605917_m1 RefSeq NM_004797); diacylglycerol-O-acyltransferase 1 *(DGAT1)* (Hs01017541_m1, RefSeq NM_012079.4); diacylglycerol-O-acyltransferase 2 *(DGAT2*) (Hs01045913_m1, RefSeq NM_032564.3) and perilipin *(PLIN)*(Hs01106925_m1, RefSeq NM_001145311.1); fatty acid-binding protein 1, 4 and 5, *(FABP4* (Hs01086177_m1, RefSeq. NM_001442.2), *FABP5* (Hs02339439_g1, RefSeq. NM_001444.1) and *FABP1* (Hs00155026_m1, RefSeq. NM_001443.1)). In liver samples we only analyzed the mRNA levels of *FABP 1, 4* and *5*. In the isolated adipocytes and SVF we analyzed the mRNA level of *FABP4*. A threshold cycle (Ct value) was obtained for each amplification curve and a ΔCt value was first calculated by subtracting the Ct value for the corresponding endogenous control cDNA from the Ct value for each sample and transcript. Fold changes compared with the endogenous control were then determined by calculating 2^−ΔCt^, and expression results are given as the expression ratio relative to *GAPDH* or *β-Actin* gene expression, according to the manufacturer’s guidelines. All samples were quantified in duplicate and positive and negative controls were included in all the reactions.

### Western Blot Assays

Total proteins from adipose tissue samples were extracted by NE-PER Nuclear and Cytoplasmic Extraction Reagents protocol (Pierce). Protein extracts (30 µg) were separated by SDS-PAGE, blotted onto a PVDF membrane and then incubated with anti-human FABP4 antibody at 1∶1000 dilution (Santa Cruz Biotechnology, CA, USA) and developed with SuperSignal® West Femto/Pico Maximum Sensitivity Substrate (Pierce, Rockford, IL, USA). Protein signals were visualized by electrochemiluminescence using Quantity One® software (Bio-Rad Laboratories). Data were normalized to the corresponding β-actin band intensities.

### Immunofluorescence

Five-micron sections of frozen adipose tissue were fixed with paraformaldehyde 4%. Staining was performed overnight at 4°C with anti FABP4 antibody at 1∶200 dilution, and anti CD68 at 1∶25 dilution, washed, and visualized using AF546 and AF488 antibodies at 1∶500 dilution (Molecular Probes Inc, OR, USA), respectively. As a negative control, the entire immunofluorescence procedure was performed in the absence of primary antibody. The slides were counterstained with DAPI.

### Immunohistochemistry

Sections of formalin-fixed paraffin-embedded adipose tissue were deparaffinised and rehydrated prior to antigen unmasking by boiling in 1 mM EDTA, pH 8. Sections were blocked in normal serum and incubated overnight with anti-human FABP4 antibody at 1∶200 dilution, and anti CD68 (Santa Cruz Biotechnology) at 1∶25 dilution. Secondary antibody staining was performed using the VECTASTAIN ABC kit (Vector laboratories, Inc. Burlingame, CA) and detected with diaminobenzidine (DAB, Vector Laboratories). Sections were counterstained with haematoxylin prior to dehydration and coverslip placement. As a negative control, the entire immunohistochemical procedure was performed on adjacent sections in the absence of primary antibody.

### Studies in Animals

Adult wild-type mice (WT) (8 males and 8 females) and ob/ob mice (8 males and 8 females) C57BL6 were purchased from Jackson Laboratory (The Jackson Laboratory, Bar Harbor, ME). All mice were housed under standard housing conditions on a 12-h light/dark cycle in humidity and temperature-controlled rooms (22°C) and fed ad libitum standard chow diet (RM3, with 11.5% of calories from fat; SDS, Essex, U.K.) during two weeks. The animals were killed after 12 hours of fasting and VAT and liver samples were immediately excised, frozen in liquid nitrogen and stored at −80°C until its analysis.

**Table 1:** Characteristics of the population, circulating FABP4 levels and *FABP4* expression levels in cohort 1.

	Cohort 1		
	Lean	Overweight	Obese
	BMI<25	25≤BMI<30	30≤BMI<35
	n = 19	n = 28	n = 15
Age (years)	51.7±16.0	57.1±15.0	57.4±12.8
Gender (male)	13 (68.4%)	16 (57.1%)	9 (60.0%)
BMI (kg/m^2^)	23.6 (22.1–24.2)	27.2 (26.5–27.9)*	32.1 (30.8–33.6)*#
Waist (cm)	83.0 (79.0–90.0)	97.0 (90.5–100.0)*	107.0 (100.0–117.2)*#
SBP (mmHg)	120 (120–127)	130 (121–140)	145 (130–160)*§
DBP (mmHg)	70 (60–80)	70 (70–80)	80 (78–90)¶
Cholesterol (mM)	5.2±1.2	4.9±1.0	5.2±0.8
HDL-C (mM)	1.5±0.5	1.3±0.3	1.4±0.3
Triglycerides (mM)	1.0 (0.7–1.6)	1.1 (0.8–1.5)	1.0 (0.7–1.3)
Glucose (mM)	4.8±0.7	5.5±0.5*	5.6±0.5*
Insulin (µIU/ml)	3.4 (2.1–6.7)	4.0 (2.8–7.2)	6.6 (4.5–16.5)¶
HOMA-IR	0.75 (0.54–1.8)	1.01 (0.52–2.09)	1.60 (1.19–4.79)¶
Non-smokers	12 (63.1%)	17 (60.7%)	9 (60%)
Current-smokers	7 (36.8%)	11(39.3%)	6 (40%)
None-Alcohol consumption	13 (68.4%)	19 (67.8%)	10 (66.7%)
Alcohol consumption^a^	6 (31.6%)	9 (32.1%)	5 (33.3%)
FABP4(ng/ml)	15.5 (12.2–23.1)	20.0 (15.6–32.9)	28.7 (21.5–44.7)¶
SAT *FABP4*mRNA	1.64 (1.4–2.3)	1.36 (1.5–1.6)¶	1.20 (0.9–1.8)¶
VAT *FABP4* mRNA	1.47 (0.6–1.8)	0.93 (0.1–1.4)*	1.47 (0.7–2.3)

BMI, body mass index; SBP, Systolic Blood Pressure; DBP, Diastolic Blood Pressure; HDL-C,

HDL cholesterol; HOMA-IR, Homeostasis model assessment of insulin resistance.

a Alcohol consumption was considered from 0.5–1 drink/day for women and 1–2 drinks/day for men.

Differences *vs*. Lean: *P<0.001;

¶ *P*<0.05. Differences *vs.* Overweight:

# *P*<0.001;

§ *P*<0.05.

Differences *vs*. Obese: ^Γ^, *P*<0.002. Data are expressed as mean and standard deviation (SD).

for normal variables or median and interquartile range (25^th^–75^th^) for non-normally distributed variables. Discrete variables are expressed as frequency.

**Table 2:** Characteristics of the population, circulating FABP4 levels and *FABP4* expression levels in cohort 2.

	Cohort 2		
	Lean	Morbid no IR	Morbid IR
	BMI<25	BMI>35	BMI>35
	n = 12	n = 14	n = 26
Age (years)	44.3±11.3	41.8±10.4	41.7±13.8
Gender (male)	6 (50%)	4 (28.6%)	7 (26.9%)
BMI (kg/m^2^)	21.2±4.7	45.4±10.3*	55.5±4.1*§
Waist (cm)	84.5±11.4	118.1±20.0*	140.3±10.9*#
SBP (mmHg)	119±14.9	133±18.9¶	147±22.4*§
DBP (mmHg)	72±15.7	82±10.6	86±17.0
Cholesterol (mM)	5.1±1.1	5.1±1.2	5.2±1.1
HDL-C (mM)	1.4±0.7	1.3±0.3	1.0±0.6
Triglycerides (mM)	1.1±0.8	1.5±0.9	1.3 (1.0–1.7)
Glucose (mM)	4.6±0.4	5.1±0.6¶	5.7±0.9*
Insulin (µIU/ml)	3.7±2.5	11.7±4.5*	30.4±13.4*#
HOMA-IR	0.97±0.45	2.62±0.92*	8.3±3.8*#
Non-smokers	9 (75%)	10 (71.4%)	19 (73%)
Current-smokers	3 (25%)	4 (28.6%)	7 (26.9%)
None- alcohol consumption	8 (66.7%)	9 (64.3%)	17 (65.4%)
Alcohol consumption^a^	4 (33.3%)	5 (35.7%)	9 (34.65)
FABP4(ng/ml)	14.9±11.9	30.8±10.1*	39.2±11.5*§
SAT *FABP4*mRNA	2.2±0.8	1.6±0.6¶	1.2±0.3^*^ §
VAT *FABP4* mRNA	2.0±0.9	1.8±0.9	1.3±0.8¶

BMI, body mass index; SBP, Systolic Blood Pressure; DBP, Diastolic Blood Pressure; HDL-C,

HDL cholesterol; HOMA-IR, Homeostasis model assessment of insulin resistance.

a Alcohol consumption was considered from 0.5–1 drink/day for women and 1–2 drinks/day for men.

Differences *vs*. Lean: *P<0.001;

¶ *P*<0.05. Differences *vs.* Morbid no IR:

# *P*<0.001;

§ *P*<0.05.

Differences *vs*. Morbid IR: ^Γ^, *P*<0.002. Data are expressed as mean and standard deviation (SD).

for normal variables or median and interquartile range (25^th^–75^th^) for non-normally distributed variables. Discrete variables are expressed as frequency.

**Table 3:** Bivariate correlations with *FABP4* adipose tissue gene expression in every depot and anthropometric and metabolic characteristics in the first cohort.

	SAT *FABP4*		VAT *FABP4*	
	R	*P value*	R	*P value*
**Population Characteristics**				
BMI	−0.323	0.003	−0.390	0.002
SBP	−0.324	0.018	–	–
HOMA-IR	−0.285	0.023	−0.332	0.030
Insulin	−0.336	0.006	−0.342	0.020
**Gene expression**				
* VAT FABP4*	0.453	<0.001	–	–
* Adiponectin*	0.821	<0.001	0.938	<0.001
* ATGL*	0.357	<0.001	0.715	<0.001
* CD36*	0.676	<0.001	0.861	<0.001
* ACSS2*	0.512	<0.001	0.526	<0.001
* DGAT1*	0.238	<0.033	0.691	<0.001
* DGAT2*	–	–	0.515	<0.001
* HSL*	0.543	<0.001	0.669	<0.001
* PLIN*	0.434	<0.001	0.808	<0.001
* PPAR*α	0.561	<0.001	0.322	0.003
* PPAR*γ	0.661	<0.001	0.798	<0.001
* FABP5*	–	–	0.478	<0.001

R, Correlation coefficient. Significant differences *P*<0.05. BMI, body mass index; SBP, Systolic Blood Pressure; HOMA-IR, Homeostasis model assessment of insulin resistance; Peroxisome proliferator-activated receptors (*PPAR*α, *PPAR*δ and *PPAR*γ); hormone-sensitive lipase (*HSL)*; adipose triglyceride lipase (*ATGL*); acyl-CoA synthetase short-chain family member 2 *(ACSS2*); CD36 molecule *(CD36);* Adiponectin *(APM)*; diacylglycerol-O-acyltransferase 1 *(DGAT1)*; diacylglycerol-O-acyltransferase 2 *(DGAT2*) and perilipin *(PLIN)*; fatty acid-binding protein 1, 4 and 5 *(FABP4, FABP5 and FABP1*).

### RNA Extraction and Gene Expression Relative Quantification in Animal Samples

The RNA isolation from VAT and liver tissue and the posterior gene expression analysis by real-time PCR in these mice samples were carried out as described above for the human samples. The commercially available and pre-validated TaqMan primer/probe sets used for real-time PCR were as follows: *GAPDH* (4352339E; RefSeq. NM_008084.2) and *β-Actin* (4352341E; RefSeq. NM_007393.1) as endogenous controls for the target gene in each reaction of samples from liver and adipose tissue, respectively, *FABP4* (Mm00445880_m1, RefSeq. NM_024406.2) and *FABP1* (Mm00444340_m1, RefSeq. NM_017399.4).

**Figure 1:**
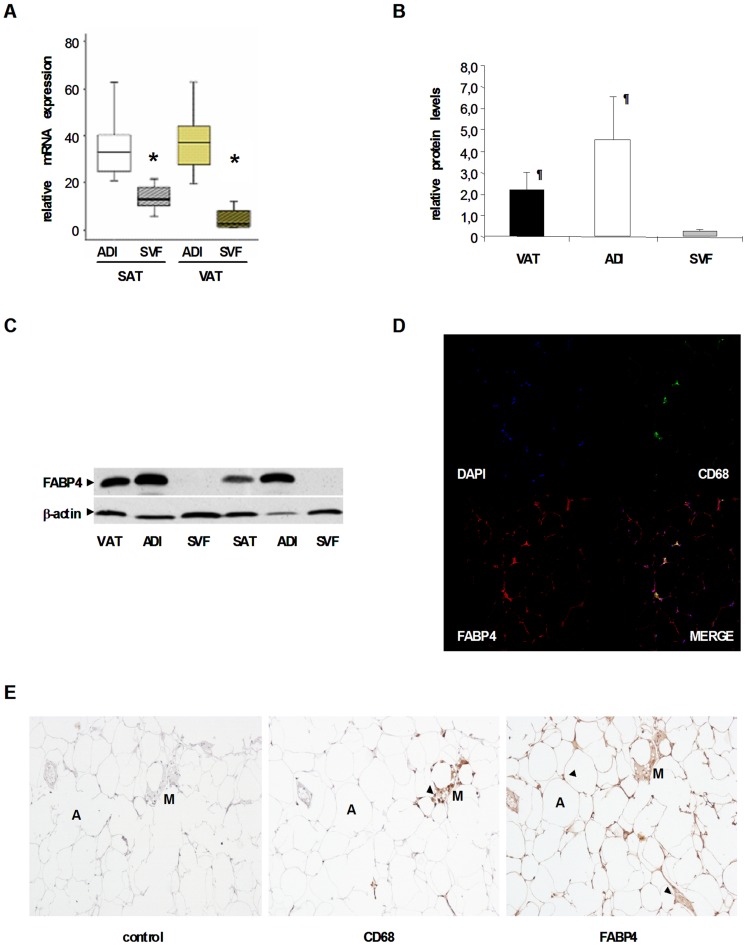
*FABP4* expression is higher in isolated adipocytes (ADI) than in the stromovascular fraction (SVF). (A) *FABP4* mRNA expression levels in ADI and SVF from n = 15 subjects with paired adipose biopsies (ADI *vs.* SVF, *P = 0.001). Data are expressed as median and IQR. (B) FABP4 protein levels in total VAT, and in ADI and SVF fractions, from n = 4 subjects. Data are expressed as mean and SD. (ADI *vs.* SVF, ^¶^ *P*<0.05) (C) Representative blot of FABP4 protein in total VAT and SAT, and in ADI and SVF fractions. (D) Immunofluorescence detection of FABP4 (red) and CD68 (green) in VAT. The counterstaining of nuclei (DAPI) is shown in blue. Images are representative of VAT collected from five subjects. (E) Immnunohistochemical detection of FABP4 (brown, right panel) and CD68 (macrophage-specific antigen, middle panel) and negative control (left panel) in VAT. Images are representative of VAT sections collected from five subjects. A: Adipocyte; M: Macrophage. Arrow-heads indicate the specific signal.

#### Statistical analysis

Statistical analysis was performed by using the SPSS software v.15 (SPSS, Chicago, IL). Normal distributed data are expressed as mean values (standard deviation, SD), and variables with a non-Gaussian distribution are expressed as median (InterQuartil Range, IQR). Discrete variables are expressed as frequency. For statistical analysis of expression variables that did not have a Gaussian distribution, values were logarithmically transformed or analyzed by non-parametrical tests. Differences between more than two groups were compared by using ANOVA with a *post hoc* Bonferroni correction. Differences between depots were analyzed by the Wilcoxon nonparametric test with a *Post-hoc* Bonferroni correction. Differences between groups according to smoking and alcohol consumption status were analyzed using the chi-square test. The Mann-Whitney U test was used to determine the differences in the VAT and SAT *FABP4* expression levels between smokers and non-smokers as well as between alcohol consumers and no alcohol consumers. *Post hoc* calculation showed that the selected sample size for the cohort 1 and the cohort 2 had a 98% and 97% power respectively for detecting a significant difference (*P*<0.05) between *FABP4* mRNA expression levels according to BMI distribution. Associations between quantitative variables were evaluated by Pearson correlation analysis or Spearman correlation for non-normally distributed variables. The independence of the associations was evaluated by linear regression analysis, adjusting for age and gender. In the first cohort in the SAT and VAT depot, the BMI, HOMA-IR index and mRNA expression of genes for lipid metabolism (*ATGL, PLIN, CD36, ACSS2, DGAT, HSL* and *FABP5 (*only for VAT depot)), *adiponectin* and *PPARs* were selected as independent variables. At the same time, in the second cohort in both adipose tissues, the BMI, plasma insulin, plasma triglyceride levels and HOMA-IR were included as independent variables. Statistical significance occurred if a computed two-tailed probability value was *P*<0.05.

**Table 4:** Anthropometric and biochemical characteristics and mRNA expression of *FABP1*, *FABP4* and *FABP5* in a subgroup of morbidly obese and morbidly obese IR patients with liver biopsies.

	Morbid no IR	Morbid IR
	n = 7	n = 10
Age (years)	43.43±8.73	44.30±8.26
Waist(cm)	123.78±13.37	125.90±9.87
Hip (cm)	140.58±8.54	144.75±9.17
BMI (Kg/m^2^ )	41.76±3.70	45.36±6.47
SBP (mm Hg)	106.42±20.75	114.24±19.31
DBP (mm Hg)	91.42±10.69	87.50±11.36
Insulin (µIU/ml)	9.15±2.16	25.15±7.26*
Glucose (mM)	5.1±0.6	5.4±0.7
Cholesterol (mM)	5.42±1.2	5.37±2.1
Triglycerides (mM)	1.4±0.4	1.3±0.8
HDL-C (mM)	1.0±0.3	1.1±0.7
LDL-C (mM)	2.6±1.0	2.2±0.7
HOMA-IR	2.09±0.54	6.03±2.18*
*FABP4* mRNA	0.78±0.48	2.28±1.27*
*FABP1* mRNA	1.01±0.20	1.63±0.75*
*FABP5* mRNA	0.25±0.19	1.65±1.28*

Data are mean ± SD;

* indicates significant differences between the means of the two groups.

(*P*<0.05). BMI, body mass index; HDL-C, HDL cholesterol; LDL-C, LDL cholesterol; HOMA-IR, Homeostasis model assessment of insulin resistance; SBP, Systolic Blood Pressure; DBP, Diastolic Blood Pressure.

**Figure 2:**
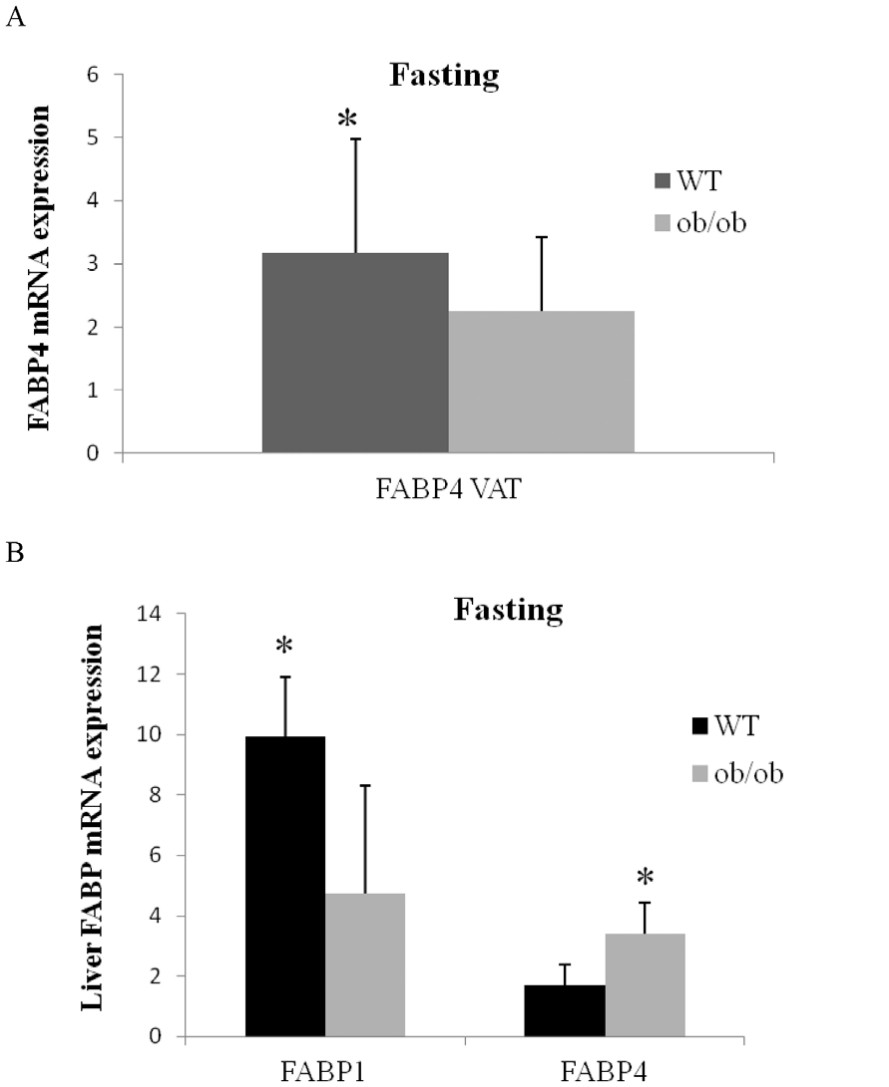
*FABP4* expression in adipose and liver tissues from mice. Fasting mRNA expression of *FABP4* visceral adipose tissue in ob/ob (n = 16) (grey bar) and WT (n = 16) (black bar) mouse. Adipose tissue expression level of the gene was normalized using *β-actin*. The results are given as the mean ± SD. ^*^indicates significant differences between the means of the two groups (*P*<0.05). (B) Fasting mRNA expression levels of hepatic *FABP1* and *FABP4* in ob/ob (n = 16) (grey bar) and WT (n = 16) (black bar) mouse. Hepatic tissue expression levels for each gene were normalized using *GADPH*. The results are given as the mean±SD. ^*^indicates significant differences between the means of the two groups (*P*<0.05).

## Results

The main clinical, anthropometrical and analytical characteristics of the two study cohorts are shown in Table 1 and Table 2.

### *FABP4* Expression in Adipose Depots

SAT and VAT *FABP4* mRNA expression levels were strongly correlated between them (R = 0.453; *P*<0.001) (Table 3). In SAT, lean subjects showed significantly higher mRNA *FABP4* expression levels than overweight and obese subjects from cohort 1. This trend was confirmed in the morbidly obese subjects from cohort 2. In VAT, *FABP4* expression levels showed a U-shaped curve, with higher levels in lean and obese subjects when compared with the overweight group (Table 1). When morbidly obese subjects were analysed, a clear down-regulation in visceral fat was observed, mainly in the MO-IR patients. There were no significant differences in the SAT and VAT *FABP4* expression levels between smoking and non-smoking either in cohort 1 or cohort 2 (cohort 1: *P* = 0.829 and *P* = 0.770 respectively; cohort 2: *P* = 0.725 and *P* = 0.721 respectively). Likewise, alcohol consumption had no significant effect on the SAT and VAT *FABP4* mRNA levels (cohort 1: *P* = 0.969 and *P* = 0.819; cohort 2: *P* = 0.480 and *P* = 0.709, respectively). No association was detected between circulating levels and subcutaneous or visceral *FABP4* gene expression. However, FABP4 plasma levels were positively associated with VAT *FABP5* gene expression (r = 0.465; *P* = 0.001). Furthermore, circulating FABP4 levels showed a significant progressive increase according to BMI, with higher levels in women and positively associated with BMI, blood pressure, HOMA-IR and FFA (data not shown).

### FABP4 mRNA/protein Expression in Total and Adipose Tissue Fractions

A selected number of subjects ranging from lean to obese phenotype were analysed for protein quantification. *FABP4* protein levels in VAT tended to be lower than in SAT. However, these differences did not reach statistical significance.

We examined whether *FABP4* expression in human adipose tissue was mainly due to mature adipocyte cells compared to other cell types from the stromovascular pool. After fractioning fresh adipose tissue, we observed that *FABP4* mRNA was significantly more expressed in the adipocyte fraction (Fig. 1A). Protein expression was confirmed by Western blot analysis (Figs. 1B and 1C) immunofluorescence (Fig. 1D) and immunohistochemistry (Fig. 1E), obtaining similar results to the mRNA expression study. Thus, FABP4 protein was basically found in mature adipocytes with a lower signal in the SVF. Immunohistochemical and immunofluorescence staining confirmed that the majority of FABP4 was localized in mature adipocytes. The signal obtained in the SVF was mainly attributed to the macrophage content surrounding the adipocytes, as was shown by CD68 co-localization immunostaining and immunofluorescence (Figs. 1D and 1E).

### Relationship between *FABP4* Gene Expression and Adipose Tissue Genes

Associations between SAT and VAT *FABP4* expression levels and genes related with lipid synthesis, FFA transport, lipolysis regulation, lipases, *PPAR* transcription factor family, *adiponectin* and FABP5 were analysed in the first cohort. The most significant bivariate associations are shown in Table 3. In order to strengthen the independence of these associations as predictors of *FABP4* expression, a multiple regression analysis model was constructed for each depot, including the above-mentioned bivariate correlations, adjusting for age and gender. In the SAT depot model, BMI, the HOMA-IR index and mRNA expression of SAT genes for lipid metabolism (*ATGL, PLIN, CD36, ACSS2, DGAT1* and *HSL*), *adiponectin* and *PPARs* were selected as independent variables. The result presented a multiple correlation coefficient (R) of 0.918, and SAT *FABP4* expression was mainly associated positively by *ATGL* (B = 0.551; *P*<0.0001) and *PPAR*α (B = 0.498; *P* = 0.001) gene expression and inversely by the HOMA-IR index (B = −0.062; *P* = 0.033). In the VAT model, BMI, HOMA-IR and the gene expression described above, including *FABP5*, were included as independent variables. The model presented an R = 0.964 and VAT *FABP4* expression was mainly associated by *adiponectin* (B = 0.302; *P*<0.0001), *ATGL* (B = 0.234; *P* = 0.027) and inversely by the HOMA-IR index (B = −0.023; *P* = 0.005).

Regarding the second morbidly obese cohort, a multiple linear regression analysis was also carried out to weight the contribution of the bivariate associations previously observed in the SAT and VAT FABP4 gene expression. The equation was also adjusted for age and gender. Thus, BMI, plasma insulin, plasma triglyceride levels and HOMA-IR were included as independent variables. BMI appeared as the only determinant, explaining nearly 50% of the *FABP4* variation in both adipose tissue depots (B = −0.384; *P*<0.0001 and B = −0.402; *P* = 0.002 in SAT and VAT, respectively).

### *FABP4* Expression in Liver

The mRNA expression of the FABP family (*FABP1*, *FABP4* and *FABP5*) was analysed in liver biopsies from a subgroup of the morbidly obese cohort (Table 4). These subjects were representative of the entire morbidly obese cohort and no differences regarding age, gender or metabolic variables except for the insulin and HOMA-IR index were observed between the groups. The MO-IR group showed a significant higher *FABP1*, *FABP4* and *FABP5* mRNA expression levels than the MO non-IR group (*P*<0.05) (Table 4).

### *FABP4* Expression in Adipose and Liver Tissues from Animal Models

*FABP4* gene expression in the VAT as well as *FABP1* and *FABP4* mRNA level in the liver were analyzed in WT and ob/ob mice subjected to fasting. We found a significantly greater *FABP4* expression in the VAT depot in WT compared to ob/ob mice (*P*<0.05). On the other hand, in the liver we found a significant two-fold increase in *FABP1* expression in WT compared to ob/ob mice (*P*<0.05). In contrast, the liver *FABP4* mRNA gene expression was significantly higher in ob/ob mice in comparison with WT mice (Fig. 2).

## Discussion

In this study we observed an inverse relationship between *FABP4* expression and obesity, with a greater down-regulation with severe IR. Interestingly, FABP4 circulating levels did not parallel adipose tissue expression. Moreover, the inverse pattern in *FABP4* gene expression observed between adipose and hepatic tissues, regarding the IR context, suggests a complementary regulatory system in which different tissues work in a balanced manner.

Several studies have linked FABP4 circulating levels with obesity and other diseases [32]–[36]. These associations suggested that FABP4 may be involved in the vascular events related with these pathologies [32]. Adipocytes are the main source of FABP4, and it would be rational to think than a higher circulating plasma protein levels, greater adipose tissue expression. However, our study demonstrates an inverse association between obesity and *FABP4* adipose tissue expression. These differences were confirmed in two separate cohorts, strengthening the validity of the observation. A previous study in morbidly obese women found no differences in *FABP4* adipose tissue expression when compared with lean counterparts; however the low number of lean subjects included may have contributed to the discrepancies [37]. Concerning FABP4 circulating levels, our results failed to find an association with adipose tissue expression.

Fisher et al., demonstrated that adipose tissue expression of FABP4 was related to circulating non esterified fatty acid levels in obese subjects, which would explain in part the insulin resistant environment observed in obesity [7]. Adipose FABP4 interacts physically with HSL promoting lipolysis [8]. In fact, we observed that *FABP4* expression was mainly determined by *ATGL*. VAT has a greater lipolytic potential than SAT, and the release of FFA from VAT directly into the portal circulation is one of the mechanisms involved in fatty liver disease and hepatic IR [38].

Viewing the *FABP4* mRNA down-regulation found in obese patients, we are tempted to speculate about a possible adipose tissue dysfunction leading to metabolic disorders. Thus, low *FABP4* expression in adipose tissue could lead to less FFA transport to β-oxidation, resulting in a FFA accumulation, which may exceed the adipose tissue storage capacity, resulting in excess fat “overspilled” to non-adipose tissues such as the liver. In the liver, the initial response is to facilitate the storage in the form of triacylglycerides but the limited capacity becomes saturated. Lipid excess produces toxic reactive species promoting lipotoxicity. These data suggest that a high adipose tissue *FABP4* expression may have a protective role, controlling the availability of FFA and their metabolites in the cytoplasm [39]–[41]. Of note, in our study the *adiponectin* adipose tissue expression appeared as a positive determinant of *FABP4* expression, a protein with a predominant role in insulin sensitivity with probable anti-inflammatory activity in metabolic diseases. These results are in line with a recent report in which data from DNA microarray-based gene expression profiling showed a progressive down-regulation of genes involved in fatty acid metabolism with increasing fat mass in patients with the metabolic syndrome [42].

Insulin resistance emerged as an important determinant of *FABP4* adipose expression. Hence, when IR increased, *FABP4* expression in adipose tissue decreased. This observation disagrees with the data obtained in several animal studies. Thus, mouse models with genetic disruption of *FABP4* showed a significant improvement in insulin sensitivity despite an increase in body weight, highlighting this protein as an important player in IR mechanisms. However, when the effect of *FABP4* knockdown in adipose tissue was studied, no effect on plasma glucose, insulin sensitivity or lipid homeostasis was observed [43]. These observations may help to explain the absence of increased *FABP4* gene expression in our patients. A possible explanation has been attributed in part to a compensatory expression increase of other FABP proteins, such as FABP5 in adipose tissue. In fact, *FABP5* mRNA was highly correlated with *FABP4* mRNA in VAT, and with FABP4 circulating levels, suggesting a possible co-regulatory role between them. Unfortunately, we have no mechanistic studies to unravel the intrinsic regulatory pathways involving IR and FABP4 regulation in human adipose tissue, but the present data suggest a positive effect of insulin-sensitivity on *FABP4* expression.

We found an significant increased mRNA expression of *FABP1*, *FABP4* and *FABP5* in the liver of morbidly obese-IR patients, which contrasts with the significantly decreased expression of FABP4 in adipose tissue that we found in the MO-IR patients compared to lean controls. Therefore, we hypothesized that decreased *FABP4* expression in adipose tissue linked to obesity could be compensated by an increased expression of *FABP4* in peripheral tissues with the onset of IR. Thus, this higher *FABP4* expression found in the liver of MO-IR patients indicates that *FABP4* expression could be a predictor of IR in morbidly obese patients. The same results were found in the animal model studied: VAT *FABP4* expression was also significantly lower in ob/ob mice as compared with WT mice. However, when liver *FABP4* expression was analyzed, we found a significant difference between WT and ob/ob mice, with a more elevated hepatic *FABP4* gene expression in ob/ob mice than in WT mice.

One of the main limitations of our study is its cross-sectional design, which does not permit to infer pathophysiological mechanisms with the observed results. Thus, the obtained results should be interpreted with caution. Moreover, we are aware that the low number of subjects analyzed for protein data and the absence of quantification of the *in vivo* fat mass depots hinder the adequate interpretation of the findings. However, protein and gene expression data were along the same lines. Another limitation was the inability to analyze liver biopsies from lean healthy subjects because of obvious ethical concerns. Finally, we have not taken into account the possible effect of lifestyle factors such as the changes in physical activity of the study subjects on the FABP4 plasma level, that previously have been described as the main predictors of FABP4 plasma modifications even after adjusting for body mass index and insulin resistance parameters.

In summary, we observed a decrease in *FABP4* expression in adipose tissue inversely associated with obesity, with the insulin-resistance status being an important determinant involved in the tissue expression. We also found an increased liver expression of *FABP4* in MO-IR patients, suggesting that adipose tissue and liver may act in a balanced manner according to the insulin resistance status. In conclusion, we propose that FABP4 may have different roles when analysed systemically or locally. Thus, a more global view is necessary to better understand its function. Further prospective experiments are required to confirm these results.
